# Personalized management of metabolic dysfunction-associated steatotic liver disease

**DOI:** 10.1016/j.iliver.2025.100207

**Published:** 2025-11-13

**Authors:** Zhen Sun, Kui Ming Chan, Haojie Jin

**Affiliations:** State Key Laboratory of Systems Medicine for Cancer, Shanghai Cancer Institute, Renji Hospital, Shanghai Jiao Tong University School of Medicine, Shanghai 200032, China; Department of Biomedical Sciences, City University of Hong Kong, Hong Kong, China; State Key Laboratory of Systems Medicine for Cancer, Shanghai Cancer Institute, Renji Hospital, Shanghai Jiao Tong University School of Medicine, Shanghai 200032, China

**Keywords:** Metabolic dysfunction-associated steatotic liver disease (MASLD), Precision medicine, Patient management

Metabolic dysfunction-associated steatotic liver disease (MASLD) is the most common liver condition globally, characterized by the accumulation of lipids within liver cells. It is closely linked to metabolic syndrome and has become a significant risk factor for cirrhosis, liver cancer, and even liver failure.^1^ In recent years, the prevalence of MASLD has risen sharply, affecting over 30% of adults worldwide, largely due to the increasing prevalence of metabolic disorders such as obesity and diabetes.^2^ As MASLD progresses, it can evolve into metabolic dysfunction-associated steatohepatitis (MASH). In 2024, the FDA approved Rezdiffra, an oral small-molecule THR-β agonist, as the first treatment for noncirrhotic MASH with moderate to advanced liver fibrosis.^3^ In 2025, the FDA also approved Wegovy injection, containing Semaglutide, a GLP-1 receptor agonist, for the treatment of MASH with moderate to advanced fibrosis.^4^ While substantial progress has been made in treating MASLD, options for non-MASH patients, who represent the majority, remain limited. Most patients still rely solely on improving diet and exercise as cornerstone treatments, lacking personalized management.

MASLD is often mistakenly perceived as simple hepatic fat accumulation. However, as understanding of its pathological mechanisms has deepened, there is much evidence that MASLD is a multifactorial disease with significant heterogeneity. Its pathogenesis, clinical presentation, disease progression, and outcomes vary widely between individuals. Many risk factors and biomarkers influencing the development of MASLD have been identified, including clinical features and genetic susceptibility. For example, MASLD can be categorized into lean and normal types based on BMI, each exhibiting different clinical presentations and prognoses.^5^ Specific genetic variants, such as the PNPLA3 rs738409 (I148M), have been linked to reduced hepatic lipid turnover, but it is not associated with ischemic heart disease.^6^ However, dichotomous classification of MASLD based on individual factors oversimplifies its complexity and cannot adequately explain the diverse heterogeneity of the disease. MASLD encompasses various biological traits and metabolic abnormalities, suggesting that comprehensive classification should consider genetic background, driving factors, clinical presentation, and patient outcomes.

Recently, several classifications aimed at decoding the heterogeneity of MASLD have been proposed, which are expected to lay the foundation for personalized management strategies in the future, such as diagnosis, risk stratification, and evaluation of new drug efficacy. In 2024, Raverdy et al. used the partitioning around medoids, an unsupervised clustering method, to identify two distinct MASLD subtypes based on six key cardiometabolic and liver-related indices: age, LDL-C, HbA1c, circulating triglycerides, BMI, and ALT.^7^ These subtypes were characterized as either cardiometabolic (CM) or liver-specific (LS). The CM cluster exhibited high HbA1c and triglyceride levels, while the LS cluster was characterized by elevated ALT levels. The proportions of these clusters in the discovery cohort were 11% for CM and 7% for LS, while in the validation cohort, the proportions were 4% and 11%, respectively. Both CM and LS clusters showed higher rates of MASH and advanced fibrosis compared to other MASLD patients. The LS cluster also displayed a stronger genetic predisposition to hepatic fat accumulation, including elevated polygenic risk scores and higher frequencies of the PNPLA3 rs738409 variant. Longitudinal follow-up indicated a high incidence of chronic liver disease in both clusters, but the CM cluster exhibited significantly higher rates of cardiovascular disease and type 2 diabetes compared to the LS cluster. Additionally, liver transcriptomic and metabolomic analyses revealed some biological differences. A parallel study by Jamialahmadi et al., based on a partitioned polygenic risk score for MASLD, identified at least two distinct types: liver-specific and systemic.^8^ Both liver-specific and systemic MASLD subtypes showed significantly higher prevalence and incidence risks for chronic liver disease, cirrhosis, and hepatocellular carcinoma, with the liver-specific subtype showing more pronounced effects. However, systemic MASLD was also associated with a higher risk of cardiovascular disease, diabetes, hypertension, and chronic kidney failure. Interestingly, the liver-specific subtype showed a significantly reduced risk of cardiovascular disease compared to systemic class.

These two studies consistently suggest that at least two types of MASLD exist: liver-specific and systemic/cardiometabolic. Therefore, the current evidence supports the notion that different clinical management strategies should be applied to different MASLD patients. For example, the systemic or CM subtype should particularly focus on preventing or monitoring the development of cardiovascular disease, and patients may benefit more from lipid-lowering agents and anti-inflammatory therapy. While the liver-specific subtype requires more attention to liver-related complications. However, further prospective trials or cohort studies are necessary. One limitation of these studies is the lack of comparison between MASLD patients and the general population. For instance, although the liver-specific subtype shows a lower cardiovascular disease risk, it remains unclear whether their cardiovascular disease risk is still higher than that of individuals without fatty liver disease. Furthermore, a major drawback is that both clinical and genetic classifications fail to encompass the majority of MASLD patients. The LS and CM classifications account for less than 20% of the total MASLD population, and in some cohorts, even less than 5%. This undermines the widespread applicability of this classification framework. A recent study by Hong et al., based on seven clinical indicators (age, AST, waist-hip ratio, monocyte-lymphocyte ratio, LDL-C, cholesterol, and BMI), used the unsupervised clustering method K-means to classify MASLD into five subtypes: metabolic-dyslipidemia, obesity, younger, inflammatory, and hepatotoxic.^9^ The hepatotoxic subtype had significantly higher risks for severe liver diseases, such as hepatocellular carcinoma and liver failure, while the inflammatory subtype showed a higher incidence of extrahepatic complications, such as stroke and coronary artery disease. As a result, both hepatotoxic and inflammatory subtypes were labeled as high-risk, accounting for approximately 34% of the total cases, with the remaining subtypes categorized as low-risk. Despite the use of different clustering methods and indicators, Hong et al.'s study also supports essential findings from Jamialahmadi et al. and Raverdy et al., showing the existence of liver-dominant subtypes, systemic impact subtypes, and less dangerous MASLD cases. However, the same issue remains; it is unclear what the characteristics and how the larger proportion of patients with so-called low-risk MASLD should be managed, and there may still be great heterogeneity within these patients. Additionally, comparisons of MASLD patients with the general population are still lacking.

Despite many unknowns, we can see that significant breakthroughs have been made in the precise treatment of MASLD (Fig. 1). Both clinical features and genetic predisposition have helped identify at least two fundamental subtypes of MASLD: “liver-dominant” and “systemic/cardiometabolic”. This represents a key step toward personalized medicine, although it may only be the first step. To transition from recognizing heterogeneity to achieving precise clinical management, prospective intervention studies will be necessary to verify whether subtype-based differentiated strategies can indeed improve clinical outcomes for MASLD. Additionally, the current classifications require further validation, as it remains unclear whether the genetic and clinical-based classifications exhibit good consistency, or whether more valuable classification markers have been overlooked. Future research should integrate multi-omics data to create a more refined map, while also identifying easily applicable biomarkers for clinical use. This will enable the translation of scientific insights into actionable decision-making tools in clinical practice. In summary, although the precise treatment of MASLD is still in the developmental stage, it is foreseeable that future therapies will become more refined and diverse, offering patients more personalized and effective management strategies.

**Fig. 1:**
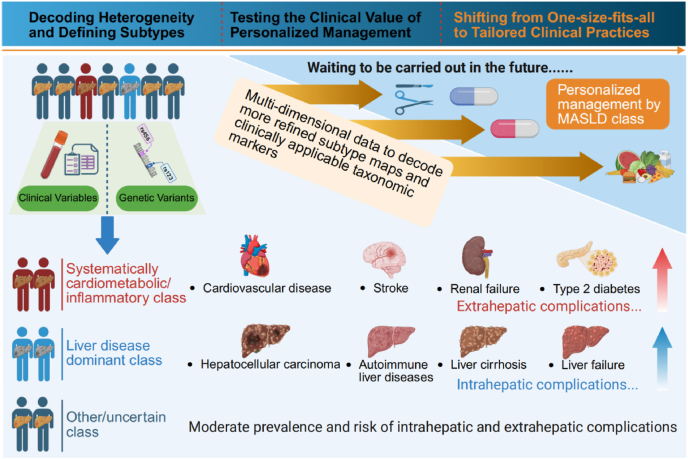
The path to personalized management of metabolic dysfunction-associated steatotic liver disease (Created in BioRender. Jin, H. (2025) https://BioRender.com/fic5a9u).

## CRediT authorship contribution statement

**Zhen Sun:** Writing – original draft. **Kui Ming Chan:** Conceptualization, Writing – review & editing. **Haojie Jin:** Conceptualization, Writing – review & editing.

## Informed consent

Not applicable.

## Ethics statement

Not applicable.

## Data availability statement

Not applicable.

## Declaration of generative AI and AI-assisted technologies in the writing process

Not applicable.

## Funding

This study was funded by the 10.13039/501100012166National Key Research and Development Program of China (2022YFC2804300).

## Declaration of competing interest

All authors and funder have no conflicts of interest to declare.
